# Metagenomes and metagenome-assembled genomes from *Onthophagus taurus*

**DOI:** 10.1128/mra.00457-25

**Published:** 2025-09-17

**Authors:** Joshua A. Jones, Armin P. Moczek, Irene L. G. Newton

**Affiliations:** 1 Department of Biology, Indiana University 1772, Bloomington, Indiana, USA; The University of Arizona, Tucson, Arizona, USA

**Keywords:** *Onthophagus*, dung beetle, microbiome, MAG

## Abstract

Shotgun metagenomic sequencing was carried out on *Onthophagus taurus* larval gut sections, female adult midguts, and pedestals (a maternally provisioned fecal pellet provided to offspring). Here, we present the raw sequencing files for five sample types and 16 annotated metagenome-assembled genomes (MAGs).

## ANNOUNCEMENT

Onthophagini dung beetles pass symbionts vertically across generations (1) and are reliant on these symbionts for normative development (2–5); however, there are little data on what function these microbes provide (6, 7). To characterize the metabolic potential of the microbiome, we sequenced bacteria from the *Onthophagus taurus* larval and adult gut sections, as well as the pedestal, a maternal fecal secretion facilitating the passing of microbiota from mother to offspring (1, 2). Here, we present five metagenomes from the larval fore-, mid-, and hindguts, the female adult midgut, and the pedestal, as well as 16 metagenomic-assembled genomes (MAGs).

Wild-caught *O. taurus* were collected near Chapel Hill, North Carolina, United States in 2019. The beetles were maintained in the lab and bred following established methods (2, 8). Pedestals (*n* = 17) were harvested from beneath the eggs, where they are deposited prior to oviposition and pooled, and DNA was extracted using a Qiagen DNeasy PowerSoil Pro Kit. Larval (*n* = 5) and adult female (*n* = 4) gut sections were dissected following surface sterilization with a 1% bleach and 0.1% triton solution and rinsed in sterile PBS. Fore-, mid-, and hindguts were dissected from both larvae and adults, pooled within the sample type, and enriched for bacterial fractions via dounce homogenization, followed by differential centrifugation (spinning out host tissue and cellular debris at 200 g before pelleting the microbial fraction at 11,000 g) (9). DNA was extracted from the enriched samples using a modified phenol: chloroform extraction. Briefly, samples were subjected to bead beating in a lysing matrix C tube using a FastPrep (2× speed 6 for 40 s), followed by proteinase K digestion and phenol: chloroform extraction (10). Resulting DNA was then sheared using a Covaris instrument to 300 bp. The NEBNext Ultra II Kit protocol was used to size-select for ~300 bp DNA fragments using Serapure beads (11) and to generate libraries. Finally, the five libraries with DNA peaks around 300 bp (pedestal, larval fore-, mid-, and hindguts, and adult female midgut) were pooled and run on a NextSeq500 sequencer using 300 paired-end cycles.

Initial sequencing resulted in 17,887,6800–18,983,874 reads from each library. Trimmomatic v.36 (12) was used to remove the adapter sequences (TruSeq3-PE) and the low-quality reads (LEADING:3, TRAILING:3, SLIDINGWINDOW:4:15, MINLEN:36). Assembly was carried out on KBase (13) using default parameters, unless otherwise noted. Sequences were assembled in metaSPAdes v1.3.4 (14) using reads pooled across the five samples, resulting in 26,789 contigs > 2 kbp and an N50 of 7,301. Contigs were then binned into metagenomically assembled genomes (MAGs) using CONCOCT v1.3.4 (15), MaxBins2 v1.1.1 (16), and MetaBAT2 v2.3.0 (17). DAS Tool v1.1.2 (18) was used to produce an optimized set of bins across the binning software. Finally, CheckM v1.4.0 (19) was used to assess bin completeness and contamination. Of the 32 assembled bins, 16 were more than 90% complete and contained less than 5% contamination. MAGs were taxonomically identified using GTDB-Tk v2.3.2 (20) and annotated using RASTtk v1.073 (21). Summary statistics and taxonomic classifications for these bins are listed in Table 1.

**TABLE 1:** Summary statistics for high-quality MAGs generated from shotgun-metagenomic reads

	MAG data:							
	NCBI quality MAGs^*a*^:							
BioSample name	JAJ_Otau_1	JAJ_Otau_2	JAJ_Otau_3	JAJ_Otau_4	JAJ_Otau_5	JAJ_Otau_6	JAJ_Otau_7	JAJ_Otau_8
BioSample identifier	SAMN41896156	SAMN41896157	SAMN41896158	SAMN41896159	SAMN41896160	SAMN41896161	SAMN41896162	SAMN41896163
Manuscript identifier	Pseudomaricurvus	Frigididesulfovibrio	Proteiniphilum	Moraxellaceae	Paludibacteraceae_B	Alphaproteobacteria	Christensenellales_A	Gemmatimonadaceae
Sequence reads	3,216,138	2,752,013	2,236,222	2,453,888	3,685,394	1,478,159	3,174,695	3,278,426
Number of contigs	261	197	275	130	238	12	139	163
N50^*b*^	17,146	23,664	10,657	31,467	34,314	204,624	35,042	33,428
L50^*b*^	55	39	67	26	26	3	22	34
GC (%)	58.50%	49.70%	49.50%	48.90%	34.20%	31.30%	41.30%	47.10%
Completeness^*c*^	94.06%	93.78%	91.08%	94.69%	97.31%	97.85%	97.78%	97.06%
Contamination^*c*^	1.13%	3.19%	0.56%	1.30%	2.69%	0.00%	2.02%	4.95%
Number of protein-coding sequences^*d*^	3,067	2,931	2,111	2,394	3,734	1,455	3,113	2,984
Domain^*e*^	Bacteria	Bacteria	Bacteria	Bacteria	Bacteria	Bacteria	Bacteria	Bacteria
Phylum^*e*^	Pseudomonadota	Desulfobacterota	Bacteroidota	Pseudomonadota	Bacteroidota	Pseudomonadota	Bacillota	Gemmatimonadota
Class^*e*^	Gammaproteobacteria	Desulfovibrionia	Bacteroidia	Gammaproteobacteria	Bacteroidia	Alphaproteobacteria	Clostridia	Gemmatimonadetes
Order^*e*^	Pseudomonadales	Desulfovibrionales	Bacteroidales	Pseudomonadales	Bacteroidales	RF32	Christensenellales	Gemmatimonadales
Family^*e*^	Cellvibrionaceae	Desulfovibrionaceae	Dysgonomonadaceae	Moraxellaceae	Paludibacteraceae	CAG-239	CAG-74	Gemmatimonadaceae
Genus^*e*^	Pseudomaricurvus	Frigididesulfovibrio	Proteiniphilum	–	QVMH01	–	–	Fen-1247
Species^*e*^	-–	–	Proteiniphilum sp012799155	–	–	–	–	–
Pedestal^*f*^	1.82%	0%	0%	1.45%	0.01%	0%	0%	0%
Larval foregut^*f*^	0.02%	0.01%	1.21%	0%	0.08%	0.04%	0%	2.12%
Larval midgut^*f*^	0%	0%	0%	0%	0.01%	0%	0%	0.01%
Larval hindgut^*f*^	0%	2.21%	0%	0%	3.12%	1.60%	2%	0%
Adult midgut^*f*^	0%	0%	0%	0%	0%	0%	0%	0%
Combined^*f*^	0.47%	0.51%	0.27%	0.37%	0.74%	0.38%	0.46%	0.51%

	MAG data:							
	NCBI quality MAGs^*a*^:							
BioSample name	JAJ_Otau_9	JAJ_Otau_10	JAJ_Otau_11	JAJ_Otau_12	JAJ_Otau_13	JAJ_Otau_14	JAJ_Otau_15	JAJ_Otau_16
BioSample identifier	SAMN41896164	SAMN41896165	SAMN41896166	SAMN41896167	SAMN41896168	SAMN41896169	SAMN41896170	SAMN41896171
Manuscript identifier	Dysgonomonas	Desulfovibrionaceae	Massilibacteroides	Negativicutes	Crocinitomicaceae	Saccharospirillaceae	Azobacteroidaceae	Saezia
Sequence reads	3,349,642	2,374,624	4,629,562	1,906,755	3,069,569	2,569,116	3,700,410	2,720,500
Number of contigs	223	290	89	262	75	39	205	241
N50^*b*^	22,836	10,265	95,405	10,061	64,598	127,277	28,629	16,548
L50^*b*^	47	77	15	57	16	7	43	47
GC (%)	36.70%	47.50%	40.00%	42.40%	35.70%	50.10%	44.20%	41.60%
Completeness^*c*^	94.81%	90.12%	99.23%	92.35%	98.92%	95.27%	96.72%	94.26%
Contamination^*c*^	0.82%	1.83%	2.26%	2.18%	1.08%	0.76%	0.00%	0.98%
Number of protein-coding sequences^*d*^	2,963	2,561	3,911	2,124	2,722	2,533	2,953	2,769
Domain^*e*^	Bacteria	Bacteria	Bacteria	Bacteria	Bacteria	Bacteria	Bacteria	Bacteria
Phylum^*e*^	Bacteroidota	Desulfobacterota	Bacteroidota	Bacillota	Bacteroidota	Pseudomonadota	Bacteroidota	Pseudomonadota
Class^*e*^	Bacteroidia	Desulfovibrionia	Bacteroidia	Negativicutes	Bacteroidia	Gammaproteobacteria	Bacteroidia	Gammaproteobacteria
Order^*e*^	Bacteroidales	Desulfovibrionales	Bacteroidales	–	Flavobacteriales	Pseudomonadales	Bacteroidales	Burkholderiales
Family^*e*^	Dysgonomonadaceae	Desulfovibrionaceae	Tannerellaceae	–	Crocinitomicaceae	Saccharospirillaceae	Azobacteroidaceae	Burkholderiaceae
Genus^*e*^	Dysgonomonas	WRHT01	Massilibacteroides	–	UBA5422	–	–	Saezia
Species^*e*^	–	–	–	–	–	–	–	–
Pedestal^*f*^	0%	0%	0.07%	0%	5.98%	8.02%	0.01%	0.02%
Larval foregut^*f*^	0.07%	0%	0.99%	0.02%	0.01%	0.01%	0.09%	0.01%
Larval midgut^*f*^	0%	0%	0.04%	0%	0.01%	0.01%	0.01%	0.05%
Larval hindgut^*f*^	2.26%	1.14%	22.36%	0.92%	0%	0%	5.65%	1.44%
Adult midgut^*f*^	0%	0%	0%	0%	0%	0%	0%	0%
Combined^*f*^	0.54%	0.26%	5.38%	0.22%	1.52%	2.02%	1.32%	0.35%

^*a*^ MAG with <90% completion and >5% contamination.

^*b*^ Measured using QUAST v4.4.

^*c*^ Completeness and contamination determined by CheckM v1.0.18.

^*d*^ Identified using RASTtk v1.073.

^*e*^ Based on the closest placement taxonomy from GRDB-Tk v1.7.0. “–” is used when MAGs could not be classified to that taxonomic rank.

^*f*^ Determined by aligning binned reads to sample assembly using Bowtie2 v2.3.2.

## Data Availability

The project discussed here has been deposited in NCBI BioProject PRJNA1117517. Included in this are the raw sequence reads from the pedestals, larval fore-, mid-, and hindguts, and female adult midguts (accession nos.: SRR29215567, SRR29215566, SRR29215565, SRR29215564, and SRR29215563, respectively). This BioProject also includes the assemblies for the 16 high-quality MAGs discussed above (accession nos.: SRR29455191, SRR29455190, SRR29455183, SRR29455182, SRR29455181, SRR29455180, SRR29455179, SRR29455178, SRR29455177, SRR29455176, SRR29455189, SRR29455188, SRR29455187, SRR29455186, SRR29455185, and SRR29455184). The assemblies and summary statistics for the16 lower quality MAGs, as well as protein annotations, have been included in a DRYAD repository (https://doi.org/10.5061/dryad.4qrfj6qn8). The accession numbers for MAG SRAs are also included in Table 1.
